# Ascorbic acid modulation by ABI4 transcriptional repression of *VTC2* in the salt tolerance of *Arabidopsis*

**DOI:** 10.1186/s12870-021-02882-1

**Published:** 2021-02-24

**Authors:** Xiamusiya Kakan, Yanwen Yu, Shenghui Li, Xiaoying Li, Rongfeng Huang, Juan Wang

**Affiliations:** 1 Biotechnology Research Institute, Chinese Academy of Agricultural Sciences, Beijing, 100081 China; 2 College of Agronomy, Xinjiang Agricultural University, Urumchi, 830052 China; 3 College of Agronomy, Henan Agricultural University, Zhengzhou, 450002 China; 4 College of Landscape and Ecological Engineering, Hebei University of Engineering, Handan, 056038 China; 5 China National Key Facility of Crop Gene Resources and Genetic Improvement, Beijing, 100081 China

**Keywords:** ABI4, Salt stress, Ascorbic acid, VTC2, ROS accumulation

## Abstract

**Background:**

Abscisic acid (ABA) plays an important role in plant abiotic stress responses, and ABA INSENSITIVE 4 (ABI4) is a pivotal transcription factor in the ABA signaling pathway. In *Arabidopsis*, ABI4 negatively regulates salt tolerance; however, the mechanism through which ABI4 regulates plant salt tolerance is poorly understood. Our previous study showed that ABI4 directly binds to the promoter of the *VITAMIN C DEFECTIVE 2* (*VTC2*) gene, inhibiting the transcription of *VTC2* and ascorbic acid (AsA) biosynthesis.

**Results:**

In the present study, we found that treatment with exogenous AsA could alleviate salt stress sensitivity of *ABI4-*overexpressing transgenic plants. The decreased AsA content and increased reactive oxygen species (ROS) levels in *ABI4-*overexpressing seedlings under salt treatment indicated that AsA-promoted ROS scavenging was related to ABI4-mediated salt tolerance. Gene expression analysis showed that *ABI4* was induced at the early stage of salt stress, giving rise to reduced *VTC2* expression. Accordingly, the abundance of the VTC2 protein decreased under the same salt stress conditions, and was absent in the *ABI4* loss-of-function mutants, suggesting that the transcriptional inhibition of ABI4 on *VTC2* resulted in the attenuation of VTC2 function. In addition, other encoding genes in the AsA biosynthesis and recycling pathways showed different responses to salt stress, demonstrating that AsA homeostasis is complicated under salinity stress.

**Conclusions:**

This study elucidates the negative modulation of ABI4 in salt stress tolerance through the regulation of AsA biosynthesis and ROS accumulation in plants.

**Supplementary Information:**

The online version contains supplementary material available at 10.1186/s12870-021-02882-1.

## Background

Ascorbic acid (AsA) plays an important role in plant growth and development [1, 2]. It is an efficient non-enzymatic antioxidant that scavenges reactive oxygen species (ROS), and not only regulates growth and development, but also modulates stress responses [3–6]. The biosynthesis of AsA and its regulatory mechanisms in plants have garnered increasing attention [7–10]. Its biosynthesis in plant leaves is regulated by light and dark [11], and it shows a circadian rhythm and responds to seasonal changes [12, 13]. The AsA content is also affected by temperature [14]. In addition, the transcriptional expression of the genes involved in AsA biosynthesis are regulated by phytohormones or secondary metabolites [15]. The L-galactose pathway is the dominant pathway of AsA biosynthesis in *Arabidopsis*. The homologous genes *VITAMIN DEFECTIVE 2* (*VTC2*) and *VTC5* encode the key GDP-L-galactose phosphorylase in this pathway, and *VTC2* plays a leading role [16–18]. Jasmonic acids (JAs) promote AsA biosynthesis through inducing the expression of *VTC2* [15, 19]. In addition to the de novo synthesis of AsA, AsA recycling also affects the AsA level [17, 20].

Salt stress limits plant growth and development, and plants have evolved a variety of adaptive mechanisms to deal with it. A large amount of ROS are produced in cells under salinity stress, which makes the antioxidant capacity of AsA more important [6, 21–23]. Previous research found that the content of H_2_O_2_ in the AsA deficient mutant *vtc1* significantly increased under salt stress [24]. The zinc-finger protein SlZF3 promoted the accumulation of AsA and enhanced plant salt stress tolerance [25]. Many studies have also reported that the exogenous supply of AsA can improve the resistance to salt stress in various plants such as corn, rice, and wheat [26–28], indicating that AsA has a positive role in salt tolerance in plants.

Abscisic acid (ABA) is known as the plant stress hormone [29, 30]. High salinity and drought dramatically increase ABA levels, which in turn induce the expression of many genes involved in stress responses [31]. Abscisic acid INSENSITIVE 4 (ABI4) functions as an important transcription factor downstream of the ABA signaling pathway [32]. The mutant *abi4* was first isolated from a screening for ABA-insensitive mutants during seed germination [33], and ABI4 has a higher transcript expression in seeds, but a lower expression at the seedling stage [34]. The ABA-deficient mutants *aba1*, *aba2,* and *aba3* show a readily-wilting phenotype under salt or drought stress, but the *abi4* mutant exhibits salt stress resistance. Plants overexpressing *ABI4* had increased salt sensitivity, because ABI4 down-regulated the Na^+^ transporter *HKT1;1* expression, indicating that a plant’s salt tolerance is related to its ability to reduce sodium accumulation in the aerial parts [35, 36]. The chloroplast development gene *AtDPG1* is involved in the salt stress response through ABI4 [37]. Therefore, ABI4 contributes to salt stress responses; however, the mechanism by which ABI4 regulates salt tolerance still needs further research.

We previously showed that ABI4 directly binds to the promoter of *VTC2*, inhibiting the transcription expression of *VTC2*, and then alleviating AsA biosynthesis [10]. ABI4 directly combines the key genes involved in ROS production and scavenging to modulate ROS metabolism during seed germination under salinity stress [38]. We found that AsA partially recovered the salt stress sensitivity of *ABI4-*overexpressing plants, which had lower AsA content and more ROS accumulation. Salt stress initially inhibited the expression of *VTC2* via promoting *ABI4* expression. Thus, ABI4-VTC2 coordinately regulates the biosynthesis of AsA under salt stress. It was revealed that the molecular mechanism of the ABI4 response to salt stress was through the regulation of AsA biosynthesis and ROS accumulation in *Arabidopsis*.

## Results

### Ascorbic acid contributes to ABI4-regulated salt stress sensitivity

**Fig. 1:**
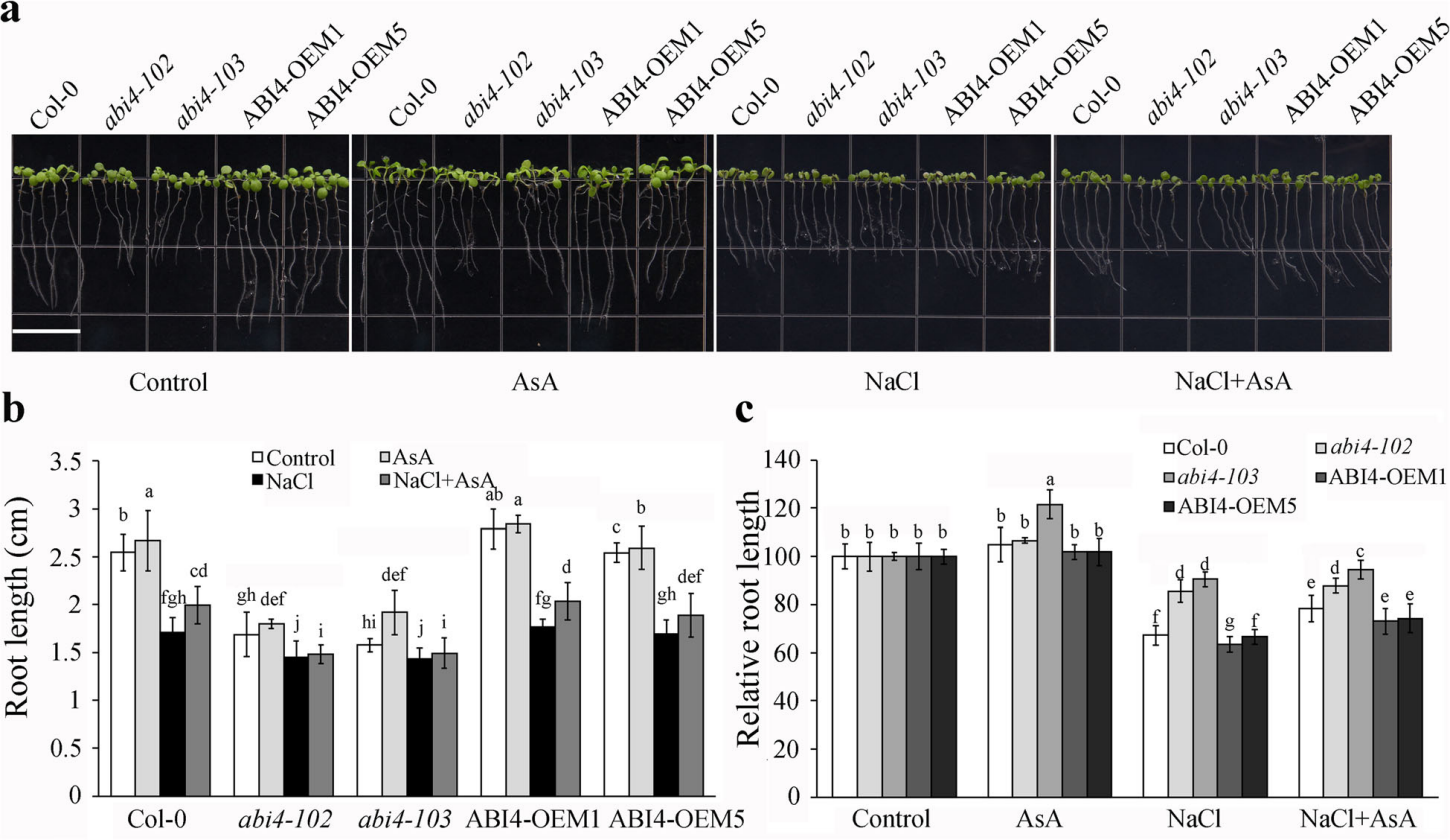
Ascorbic acid (AsA) alleviates the inhibitory effect of salt stress on root growth in ABI4-overexpressing seedling lines. **a** Seedling growth phenotype of the Col-0, *abi4–102*, *abi4–103* and *ABI4*-overexpressing lines under 100 mM NaCl treatment with or without 40 μM AsA. 3-day-old seedlings were transferred to 1/2 MS medium containing salt with or without AsA and grown for another four days. Bar = 1.4 cm. **b** The root length of Col-0, *abi4–102*, *abi4–103* and *ABI4*-overexpressing lines under 100 mM NaCl treatment with or without 40 μM AsA. Values are means ± SD (*n* = 30). **c** The relative root length of Col-0, *abi4–102*, *abi4–103* and *ABI4*-overexpressing lines under 100 mM NaCl treatment with or without 40 μM AsA compared with those under control conditions. Values are means ± SD *n* = 3. The root length of all seedlings under control conditions were normalized as 100. Different letters indicate statistically significant differences among the indicated data (*P* < 0.05, ANOVA with Tukey’s test)

Ascorbic acid has an important effect on the scavenging of the accumulated ROS under salt stress to enhance the tolerance of plants [6]. It was demonstrated that *abi4* mutants exhibit increased salt stress resistance [35]. We previously showed that ABI4 inhibits AsA biosynthesis [10], so we further analyzed the role of AsA in the ABI4-mediated response to salt stress by supplying exogenous AsA. 3-day-old seedlings of Col-0, two recessive knockout alleles of *ABI4* (*abi4–102* and *abi4–103*) and two *ABI4*-overexpressing lines with C-terminal truncated peptide lines (OEM1 and OEM5) [39] were transferred to 1/2 MS medium containing 40 μmol AsA only or 100 mM NaCl supplied with or without AsA. The root length of the seedlings was observed after they were cultured for another four days. The different genotypes exhibited a little increased root length with AsA treatment only in abi4–103 and different root length reductions under the salt stress treatment (Fig. 1a). The statistical analysis indicated that, compared to Col-0 under normal growth conditions, the root lengths of the *abi4* mutants were shorter. The root length of *abi4–103* and *abi4–102* were less inhibited under salt stress compared with Col-0, while ABI4-OEM1 and ABI4-OEM5 displayed the same trend as the Col-0 seedlings. Additionally, supplementation with AsA under salt stress partially recovered the root length in Col-0 and ABI4-OEM, but this effect was absent in the *abi4* mutants, possibly due to low concentrations of exogenous AsA (Fig. 1b and c). These results demonstrated that AsA could partially recover the ABI4-mediated salt inhibition on root growth.

**Fig. 2:**
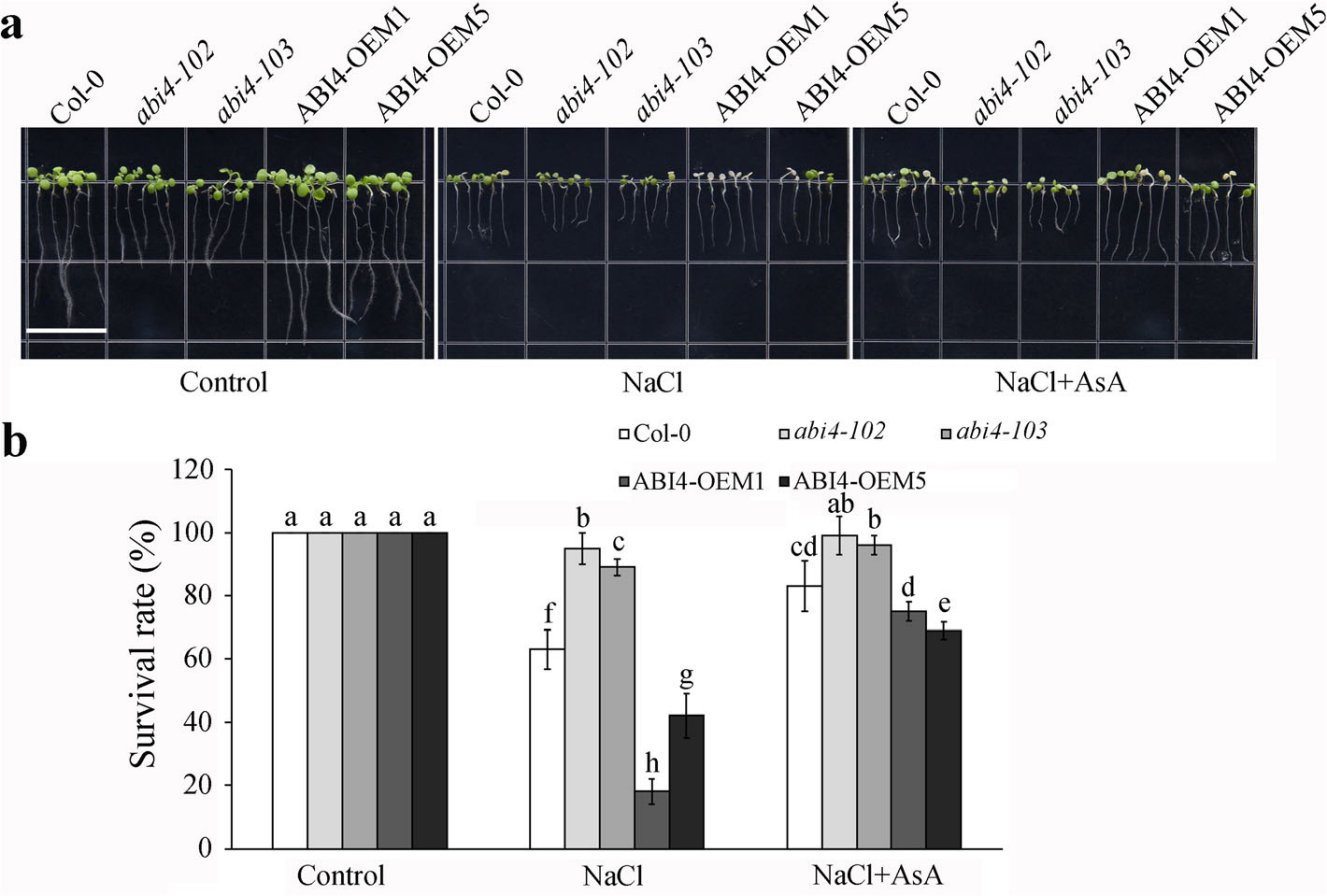
Ascorbic acid (AsA) improves the high salinity tolerance of *ABI4*-overexpressing lines. **a** The survival phenotype of different *ABI4* genotypes after 150 mM NaCl treatment (with or without 40 μM AsA). 3-day-old seedlings were transferred to 1/2 MS medium containing salt with or without exogenous AsA and grown for another four days. Bar =1.4 cm. **b** The survival rate of different *ABI4* genotypes after 150 mM NaCl treatment (with or without 40 μM AsA). Values are means ± SD (*n* = 3). Statistically significant differences are indicated by different letters (*P* < 0.05, ANOVA with Tukey’s test)

High salinity stress causes leaves to whiten and even die in *Arabidopsis* [21] We observed the seedling survival rate under 150 mM NaCl with or without supplementation of AsA. The occurrence of albinism in the *abi4* mutant seedlings was reduced, while it was higher in the OEM transgenic lines under the 150 mM NaCl treatment (Fig. 2a). Supplementation with AsA significantly enhanced the tolerance of all genotypes to salt stress. The survival rates of OEM1 and OEM5 were about 18 and 42% under NaCl treatment, respectively, while the survival rates increased up to about 75 and 69% with exogenous AsA, which were significantly improved than the Col-0 seedlings (Fig. 2b). Supplementation with AsA also improved the survival rate of the *abi4* mutants to high salinity stress. These results indicated that the ABI4-inhibited AsA synthesis mediated the salt stress sensitivity of the *abi4* mutants and ABI4-OEM seedlings.

### ABI4 regulates ascorbic acid biosynthesis and reactive oxygen species scavenging under salt stress

**Fig. 3:**
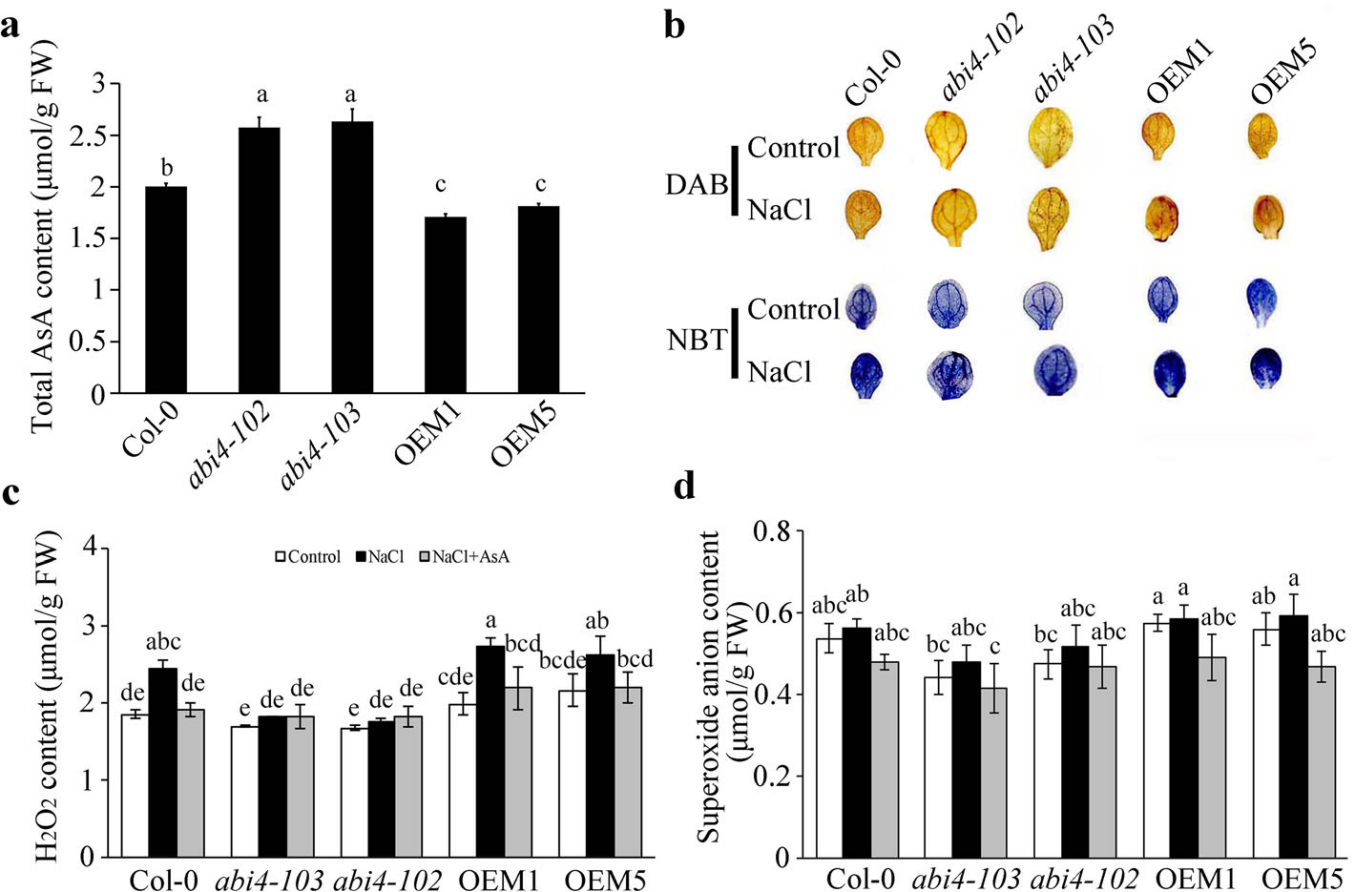
ABI4 confers to ascorbic acid (AsA) biosynthesis and reactive oxygen species (ROS) accumulation under salt stress. **a** AsA contents in Col-0, *abi4–102*, *abi4–103* and *ABI4*-overexpressing lines under the 100 mM NaCl treatment. **b** Diaminobenzidine (DAB) and nitroblue tetrazolium (NBT) staining of ROS in Col-0, *abi4–102*, *abi4–103* and *ABI4*-overexpressing seedlings with or without the salt treatment. Measurements of **c** H_2_O_2_ and **d** O_2_^−^ in Col-0, *abi4–102*, *abi4–103* and *ABI4*-overexpressing lines with or without the salt treatment. 7-day-old seedlings were treated with 100 mM NaCl (with or without 40 μM AsA) for 24 h. Values in **a**, **c** and **d** are means ± SD (*n* = 3). Statistically significant differences are indicated by different letters (*P* < 0.05, ANOVA with Tukey’s test). FW, fresh weight

Ascorbic acid plays an important role in scavenging ROS, which significantly improves plants’ tolerance to stress [40–42]. We previously found that *abi4* accumulated less ROS through ABI4 negatively regulating AsA synthesis [10]. We measured the AsA and ROS contents in 7-day-old seedlings under salt stress in the current study. The results showed that the AsA contents under the salt treatment were higher in the *abi4–103* mutants and lower in the ABI4-OEM1 and ABI4-OEM5 seedlings than in Col-0 (Fig. 3a). We then compared the ROS contents in the *ABI4* knockout mutants and the OEM transgenic plants under normal conditions and the NaCl treatment with or without AsA. Based on staining with diaminobenzidine (DAB) or nitroblue tetrazolium (NBT), H_2_O_2_ and O_2_^−^ accumulated more in the leaves of OEM1 and OEM5 than Col-0 and *abi4* mutants under both conditions. In contrast, the H_2_O_2_ and O_2_^−^ contents in the *abi4–103* leaves were significantly lower than in the Col-0 plants (Fig. 3b). The quantitative analysis showed that the level of H_2_O_2_ increased significantly with the salt stress treatment in all genotypes except the *abi4* mutants, and the ABI4-OEM seedlings accumulated more H_2_O_2_ than the Col-0 under the salt stress treatment (Fig. 3c). The content of O_2_^−^ was lower in the *abi4–103* mutants than that in the Col-0 and ABI4-OEM plants under normal and salt stress conditions, and with the addition of exogenous AsA under salt stress treatment, O_2_^−^ accumulation was obviously decreased in the ABI4-OEM plants (Fig. 3d). These results indicated that the AsA levels in *ABI4* mutants and overexpressing plants contribute to the ROS accumulations under the salt stress treatment.

### Salt stress negatively regulates *VTC2* expression through inducing *ABI4* expression

**Fig. 4:**
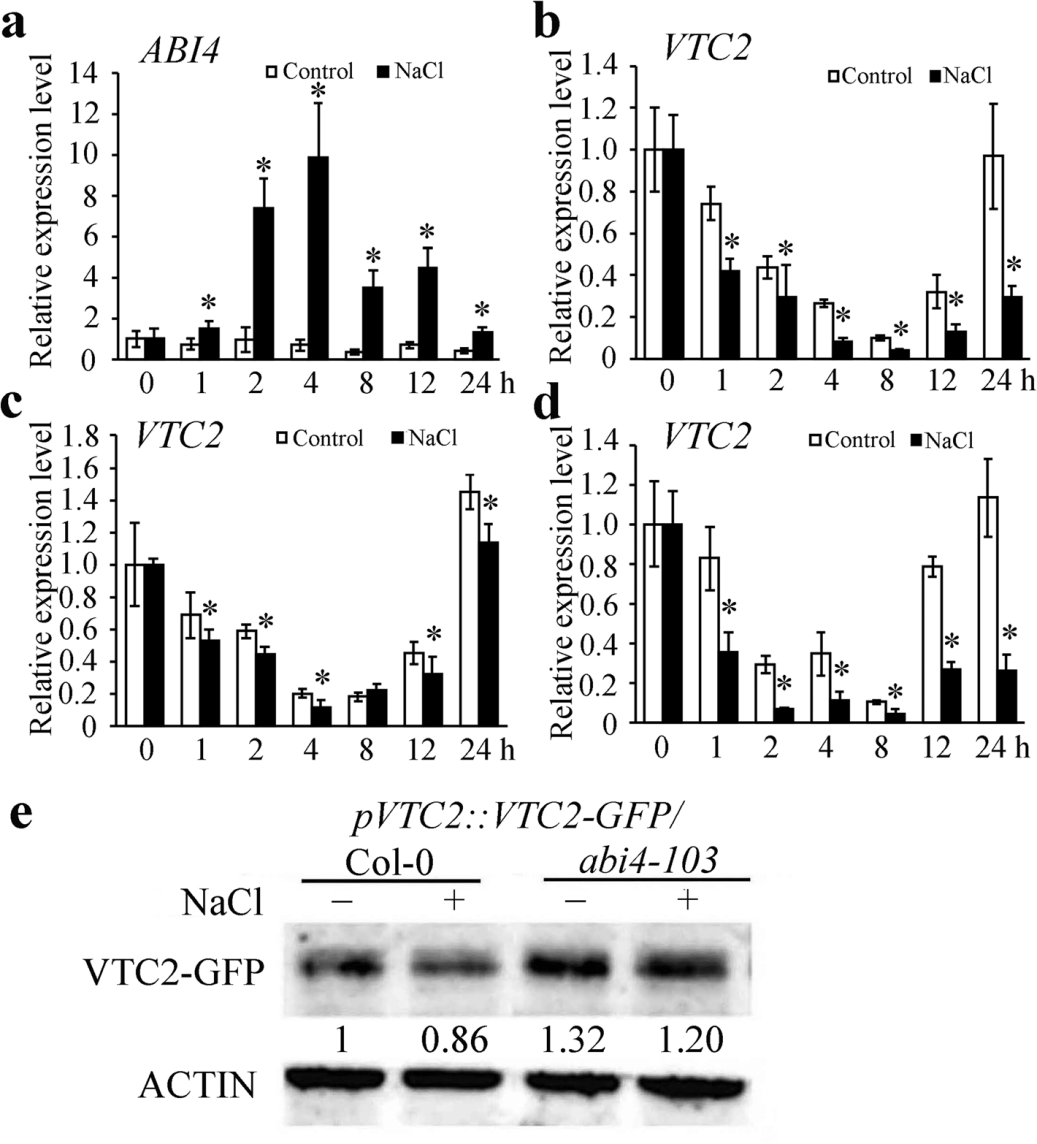
Effect of salt stress on the expression of *ABI4* and *VTC2*. The expressions of *ABI4* in Col-0 **a**, *VTC2* in Col-0 **b**, *VTC2* in *abi4–103* **c**, *VTC2* in ABI4-OEM1 **d** seedlings under 200 mM NaCl were analyzed by qPCR. After normalizing to the internal control *ACTIN*, the transcript levels of *ABI4* and *VTC2* at the indicated treatment times were compared with that at 0 h, which was normalized as “1”. Error bars represent the SD from three biological replicates. Statistically significant differences are indicated by star symbol (P < 0.05, Mann-Whitney U test). **e** Comparison of VTC2 protein levels in Col-0 and *abi4* seedlings. The numbers under the bands indicate the relative light intensity of bands, the detected band in *pVTC2::VTC2-GFP*/Col-0 without NaCl treatment was normalized as “1”. Total proteins were extracted from *pVTC2::VTC2-GFP*/Col-0 and *pVTC2::VTC2-GFP*/*abi4–103* seedlings under 100 mM NaCl treatment for 24 h and detected using anti-GFP antibodies. The detection of ACTIN was used as loading control

It was demonstrated that salt stress induced *ABI4* expression in *Arabidopsis* seedling shoots [36]. Our previous research indicated that ABI4 inhibits *VTC2* expression [10]. Therefore, it is necessary to further detect the gene expression of *VTC2* under salt stress. The expression of *ABI4* was quickly and significantly induced with 2 h until to 24 h of salt treatment, as measured by a quantitative polymerase chain reaction (qPCR) analysis (Fig. 4a). The *VTC2* expression was obviously inhibited at the beginning of 4 h of salt stress in Col-0 (Fig. 4b), while the inhibition was impaired in *abi4–103* mutants (Fig. 4c) and slightly increased in ABI4-OEM1 (Fig. 4d), suggesting that the inhibited *VTC2* transcription at the early stage of salt stress is partially ABI4 dependent.

The VTC2 protein levels in the Col-0 and *abi4* mutants under the salt stress treatment were analyzed to verify the downregulated *VTC2* expression related to the enzymatic function of VTC2. We detected the VTC2-GFP fusion protein driven by the *VTC2* promoter in the background of the Col-0 and *abi4–103* mutant treated with or without NaCl by using anti-GFP antibodies. We found that the VTC2-GFP protein accumulated more in *abi4–103* than in Col-0, which was decreased by salt stress in the Col-0 background, but less change occurred in the *abi4–103* mutant background (Fig. 4a). These results implied that the ABI4 transcriptional suppression of *VTC2* conferred to decreased AsA contents and accumulated ROS at an early stage of salt stress.

**Fig. 5:**
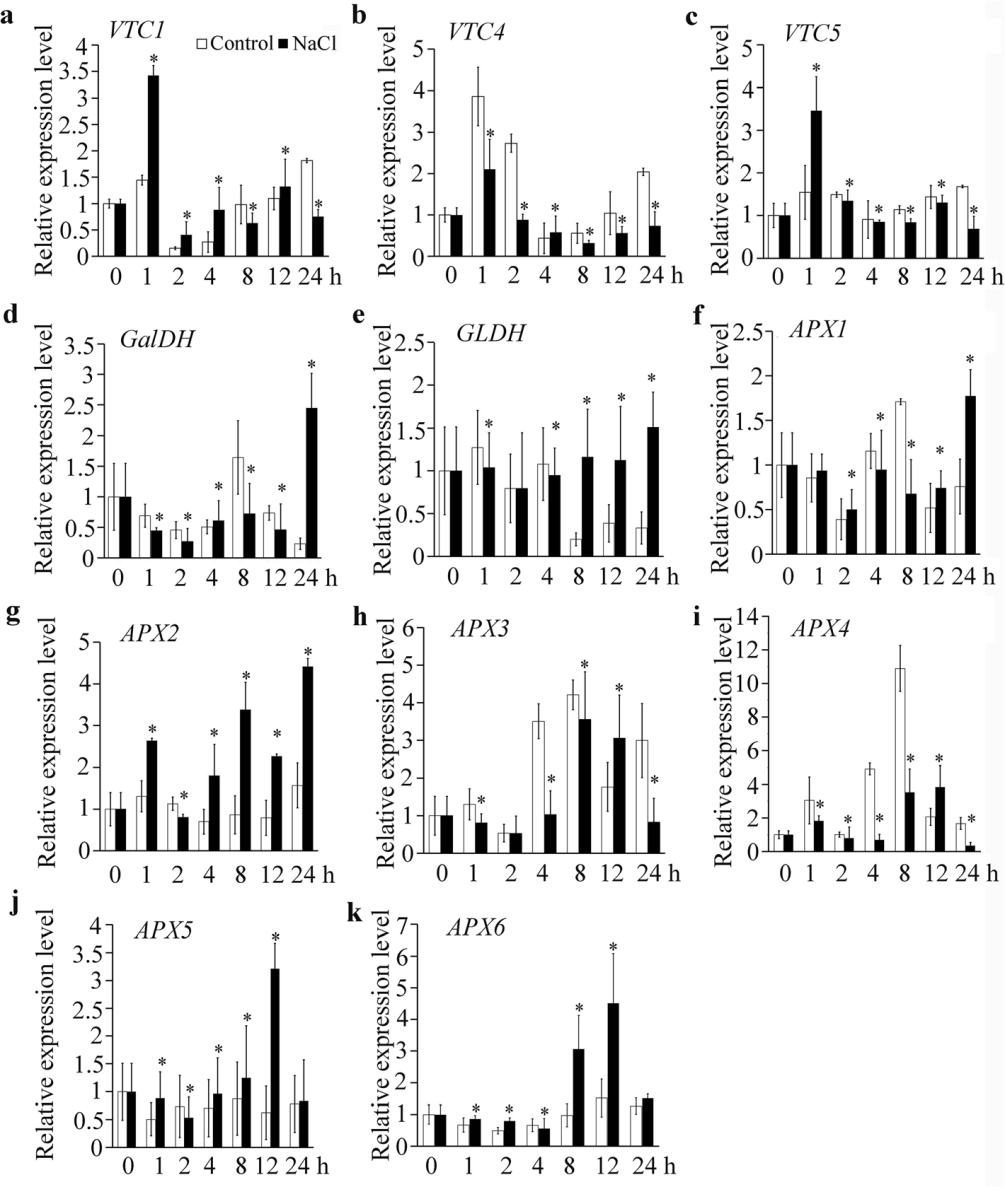
Effect of salt stress on the expression of genes involved in the L-gal pathway of ascorbic acid (AsA) synthesis and encoding ascorbic acid peroxidase (APX). The gene expressions of *VTC1*, *VTC4, VTC5, GaIDH, GLDH, APX1, APX2, APX3, APX4, APX5,* and *APX6* in Col-0 seedlings under 200 mM NaCl were analyzed by qPCR. After normalizing to the internal control *ACTIN*, the transcript levels at indicated treatment times were compared with that at 0 h, which was normalized as “1”. Error bars represent the SD from three biological replicates. Statistically significant differences are indicated by star symbol (*P* < 0.05, Mann-Whitney U test). 7-day-old seedlings were treated with 200 mM NaCl for 24 h

We also detected the expression of other genes involved in AsA synthesis and recycling under salt stress. The transcription levels of *VTC1* and *VTC5* significantly increased with 1 h of salt treatment (Fig. 5a and c). The other key genes involved in the L-gal pathway of AsA synthesis were downregulated by salt (Fig. 5b, d, and e). In the first 24 h of salt stress, the genes encoding ascorbic acid peroxidase (APX) were upregulated by salt stress except for *APX3* and *APX4* (Fig. 5F-5K). The results showed that the decreased AsA contents were related to AsA biosynthesis and recycling at the early stages of salt stress.

### ABI4 modulates salt tolerance in coordination with VTC2

**Fig. 6:**
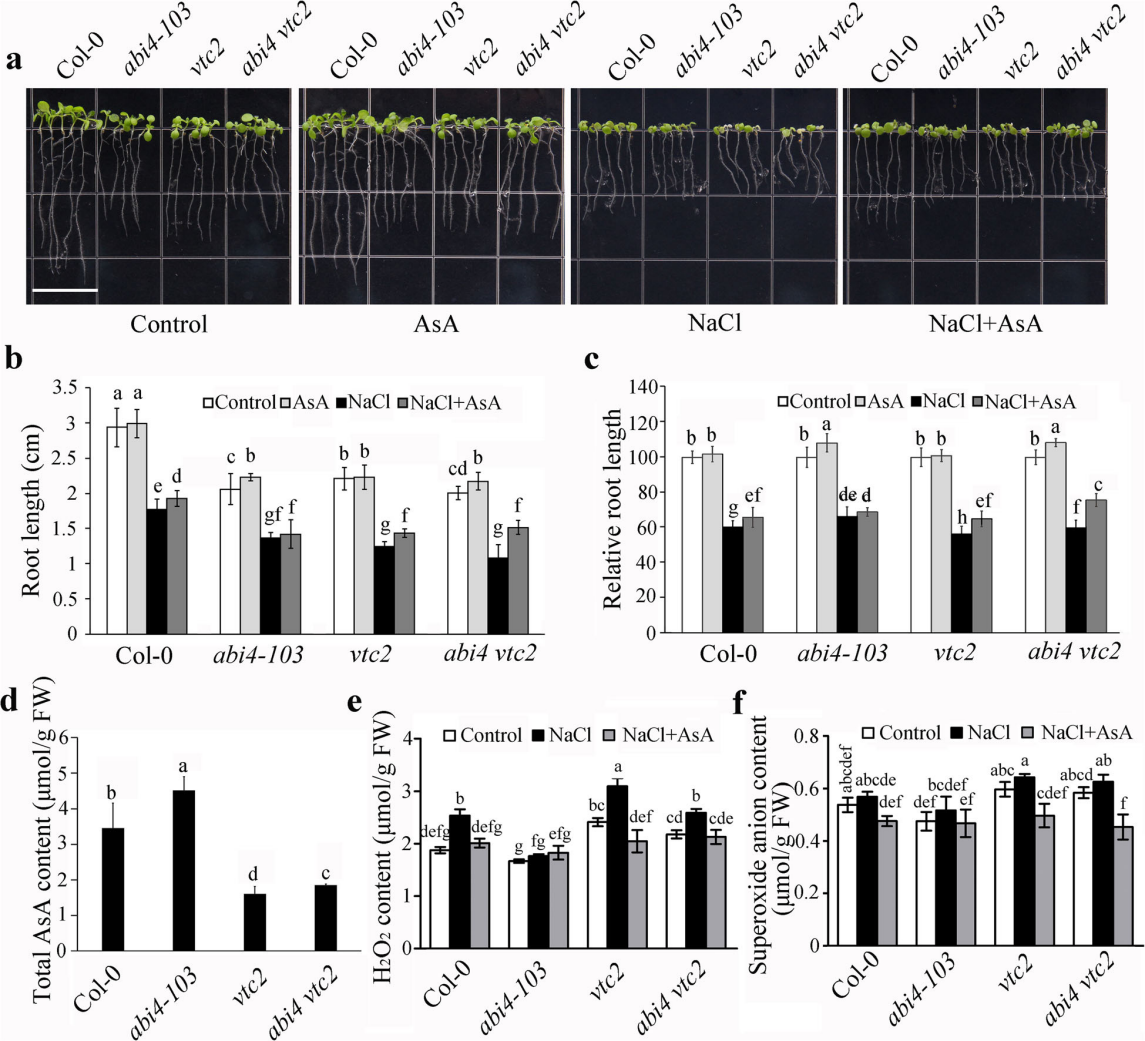
*VTC2* functions downstream of *ABI4* in modulating seedling growth under salt stress through modifying ascorbic acid (AsA) biosynthesis and reactive oxygen species accumulation. **a** Seedling growth phenotype of *abi4–103*, *vtc2* and *abi4 vtc2* mutants under 100 mM NaCl treatment with or without 40 μM AsA. Bar = 1.4 cm. **b** Root length of *abi4–103, vtc2* and *abi4 vtc2* mutants in (A). **c** Relative values of root length in (B). The root length of Col-0 and all mutants under normal growth conditions was normalized as “1”. **d** Contents of AsA in Col-0, *abi4–103*, *vtc2* and *abi4 vtc2* under the salt stress treatment. **e** H_2_O_2_ and **f** O_2_^−^ in Col-0, *abi4–103*, *vtc2* and *abi4 vtc2* under the salt stress treatment with or without 40 μM AsA. 7-day-old seedlings were treated with 100 mM NaCl (with or without 40 μM AsA) for 24 h. Values are means ± SD (n = 3). Statistically significant differences are indicated by different letters (P < 0.05, ANOVA with Tukey’s test). FW, fresh weight

According to the *ABI4* and *VTC2* gene expression responses to salt stress, we measured the salt tolerance of *abi4 vtc2* double mutants to confirm their coordinative work. The double mutants displayed shorter root length compared to Col-0 under normal conditions, and the root growth inhibition in *abi4 vtc2* mutants was more than that in *abi4–103* and less than that in *vtc2* under the salt treatment (Fig. 6a, b, and c). Further statistical analysis indicated that the root lengths of the *abi4–103*, *vtc2* and *abi4 vtc2* mutants were same under NaCl treatment, which is recovered partially with the addition of exogenous AsA (Fig. 6b). The AsA contents in the *vtc2* and *abi4 vtc2* mutants under the salt stress treatment were much less than in Col-0 (Fig. 6d), and the ROS accumulation in *abi4 vtc2* was higher than in *abi4* under salt stress (Fig. 6e and f). These results demonstrated that *VTC2* was downstream of *ABI4* in modulating AsA biosynthesis and ROS accumulation under salinity stress.

## Discussion

It was reported that ABI4 regulated plant salt tolerance and the *abi4* mutant exhibited salt stress resistance, which was related to less sodium accumulation in plant shoots [35]. However, based on our previous study showing that ABI4 mediates the cross-talk of ethylene and ABA in AsA biosynthesis [10], it is unclear whether increased AsA contents confer to the enhanced salt tolerance of the *abi4* mutants. Salinity damage to plants is caused in part by salt-induced ROS [43]. Whether the mechanism of how ABI4 regulates plant salt tolerance is related to oxidative stress has not been reported. In the current study, we uncovered the modulation of ABI4-mediated AsA biosynthesis in the salt tolerance of *Arabidopsis*. Salt stress-induced *ABI4* expression caused decreased *VTC2* transcription, leading to a reduction of AsA biosynthesis and increased ROS contents during the early stage of stress, which gave rise to retarded seedling growth. Therefore, AsA contributed to the ABI4-mediated salt stress sensitivity.

The accumulated ROS in plants has an important effect on plant growth and development [6, 44, 45]. If the ROS produced during the early stage of stress is not scavenged in time, it will cause severe cell damage. Studies have shown that reduced ROS scavenging ability can significantly impair the tolerance of plants to salt stress [46]. The *abi4–103* mutants accumulated more AsA in vivo, which can reduce the damage of ROS, while the ABI4-OEM plants were more sensitive to salt due to decreased AsA contents. Further evidence of ABI4 regulating AsA in scavenging ROS and contributing to salt tolerance was observed in the recovery of salt tolerance through the addition of AsA.

A key enzyme in the AsA synthesis pathway in plants is VTC2, and its activity has an important effect on AsA synthesis [9, 47]. The activity of VTC2 in vivo can be regulated in transcription and post-transcription levels [16]. Combining the suppressed *VTC2* expression and decreased VTC2 protein level at the early stage of salt stress, the abundance of VTC2 protein was more in the *abi4* mutants than in Col-0, suggesting that the enzymatic activity of VTC2 was enhanced in *abi4* mutants and helped to improve AsA biosynthesis under salinity stress. The contents of AsA and ROS in the *abi4* mutants were in accordance with its salt tolerance, demonstrating that the modulation of ABI4 in AsA biosynthesis played an important role in salt stress responses. It makes sense that AsA biosynthesis was downregulated during the early stage of salt stress, which gave rise to ROS accumulation and retarded the growth of the seedlings. With the accumulation of ROS under salinity stress, the genes encoding the last two steps of AsA biosynthesis, *GalDH* and *GLDH*, were induced at 24 h of the salt stress treatment to promote AsA biosynthesis and scavenge ROS. Thus, the enzymes involved in AsA biosynthesis may have complicated regulation modes in response to salinity stress.

Meanwhile, we also detected the transcription levels of the *APX* genes in the AsA recycling pathway, which are pivotal in keeping ROS homeostasis [48]. APXs in *Arabidopsis* are classified on the basis of their sub-cellular localization, three cytosolic (APX1, APX2, APX6), three microsomal (APX3, APX4, APX5) and two chloroplastic types (stromal sAPX, thylakoid tAPX) isoforms [49]. In this study, most *APX* genes had no significant changes in the first 2 h of salt stress except for *APX2*. The genes *APX1*, *APX2*, *APX5*, and *APX6* were induced at 12 h or 24 h of the salt stress treatment, while *APX3* and *APX4* were downregulated at 24 h of the treatment, which suggested that the reduced AsA contents at early stage of salt stress have direct impact on the peroxisome-localized APX3 [50] and chloroplast-localized APX4 [51], and the simultaneous ROS accumulation could be eliminated by cytosolic APXs firstly. These results are consistent with the previous conclusions that the APXs localized in different organelles have distinct functions [52]. These results showed that the AsA-mediated elimination of ROS under salt stress still requires further study.

Due to the negative regulation of *ABI4* on *VTC2*, the similarity of the salt sensitivity of the *abi4 vtc2* double mutant with *vtc2* indicated that *VTC2* is downstream of *ABI4* in regulating salt tolerance. The response of *ABI4* expression to salt stress resulted in decreased *VTC2* expression, causing reduced AsA biosynthesis and increased ROS accumulation. The increased salt tolerance of the *ABI4* loss-of-function mutants was closely related to the higher level of AsA.

## Conclusions

**Fig. 7:**
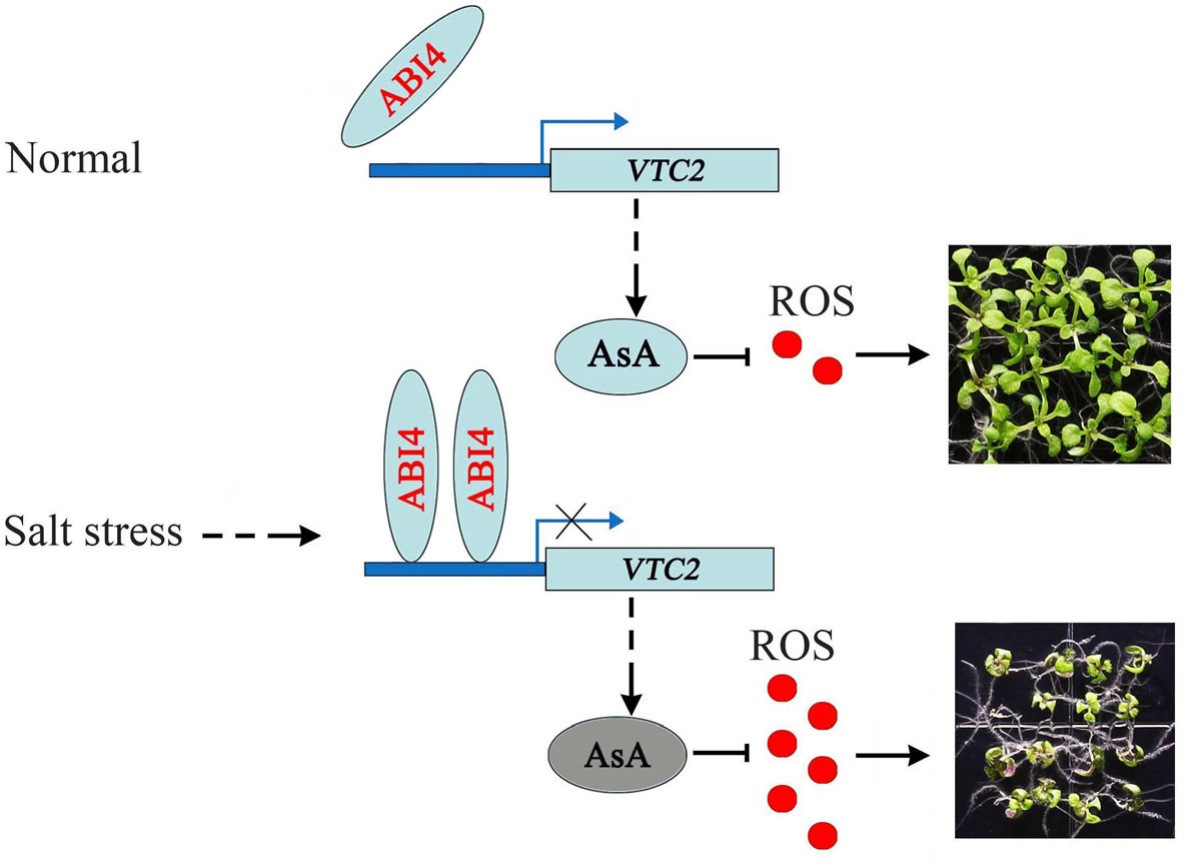
ABI4 and VTC2 coordinately regulate salinity-inhibited seedling growth through ascorbic acid (AsA) scavenging reactive oxygen species (ROS). Under normal conditions, the lower levels of the expression of ABI4 give rise to higher levels of VTC2 expression and AsA contents to scavenge ROS. Under salinity stress, ABI4 is induced and suppresses VTC2 expression. The decreased expression of VTC2 causes reduced AsA production at the early stage of salt stress; thus, more ROS is accumulated in plants. High levels of ROS can cause seedling growth inhibition or death

In conclusion, our findings offer insights into how ABI4 modulates salt-inhibited seedling growth in coordination with VTC2. The expression of *ABI4* was promoted when plants were exposed to salt stress, and then ABI4 bound to the promoter of *VTC2* to suppress its expression. Therefore, the reduced expression of *VTC2* led to reduced AsA production and increased ROS accumulation, ultimately inhibiting seedling growth (Fig. 7). Under normal conditions, the lower expression levels of *ABI4* gave rise to higher *VTC2* expression and AsA contents for scavenging ROS. Under salinity stress, *ABI4* was induced and suppressed *VTC2* expression. The decreased expression of *VTC2* reduced AsA production during the early stage of salt stress; thus, more ROS accumulated in the plants. The high level of ROS would cause seedling growth inhibition or death. Thus, the synergistic regulation of the ABI4-VTC2 module at the early stage of salt stress led to the accumulation of ROS and the inhibition of seedling growth.

## Methods

### Plant material and growth conditions

*Arabidopsis thaliana* ecotype Columbia (Col-0) was used for all experiments in this study. The *abi4–103*(CS3838), *abi4–102* (CS3837) were obtained from the Arabidopsis Biological Resource Center. The *vtc2* mutant, ABI4-OEM1, ABI4-OEM5 and pVTC2::VTC2-GFP transgenic plants have been previously described [10, 40, 53]. The plants were grown on 1/2 MS medium [54] containing 0.4% (w/v) phytagel under a 16-h-white light /8-h-dark cycle at 22 °C.

### Genetic manipulation

The double mutant *abi4 vtc2* was generated by crossing *abi4–103* with *vtc2*, and the F2 progeny from the crosses were subsequently screened by sequencing. Information about the primers used is summarized in Supplemental Table 1.

### Salt stress assays

The 3-day-old seedlings were transferred to the 1/2 MS medium containing 100 mM or 150 mM NaCl supplied with or without 40 μM AsA. Photos were taken after four days of the salt treatment, followed by a statistical analysis of the root length and survival rates. For the gene expression detection, the 7-day-old seedlings were transferred to clean filter paper and treated with 1/2 MS liquid culture with 200 mM NaCl.

### Measurement with ascorbic acid contents

For the standard curve, 0.175 g ascorbic acid was transferred into a 1.5 ml centrifuge tube and 1 ml of 6% perchloric acid (HClO_4_) was added to prepare the 1 mM AsA solution. The AsA mother liquor was diluted with 6% HClO_4_ to 0.1 μM, 0.2 μM, 0.4 μM, 0.6 μM, 0.8 μM, and 1 μM AsA standard solutions. Then, each 200 μl standard sample was transferred into a 2 ml centrifuge tube with 1800 μl 0.2 M sodium butyrate buffer (pH =12.7). The absorption value of each sample was measured at A_265_.

The measurements of the AsA contents were conducted as previously described [28]. The 7-day-old seedlings were treated with 100 mM NaCl for 24 h, then about 0.1 g samples were added into a 2 ml centrifuge tube and frozen in liquid nitrogen, crushed into a powder with plant crusher, had 1 ml 6% HClO_4_ added and were mixed well on ice for 5 min. The samples were centrifuged at 12,000 rpm for 10 min. Then, 200 μl of the supernatant was transferred to a new tube containing 1800 μl 0.2 M sodium succinate buffer (pH = 12.7). The OD1 (Optical Density 1) was measured at 265 nm by a spectrophotometer. Another 200 μl of the supernatant was added into another tube containing 1800 μl 0.2 M sodium succinate buffer (pH = 12.7) and 4 U ascorbic acid oxidase (Sigma). After being mixed and left at room temperature in the dark for 20 min, the OD2 was measured at 265 nm. Meanwhile, another 200 μl of the supernatant was added into a tube containing 1800 μl 0.2 M sodium succinate buffer (pH = 12.7) and 60 μl 1 M dithiothreitol (DTT). After being mixed and left at room temperature (25 °C) in the dark for 30 min, the OD3 was measured at 265 nm by a spectrophotometer. The concentration of the reduced or oxidized form of AsA was calculated by the value of OD1-OD2 and OD3-OD1 according to the standard curve, respectively. The sum of the two was the total AsA concentration of each sample.

### Reactive oxygen species staining

The seedlings treated with water (control) or salt (NaCl) were placed in tubes with 2 ml of the DAB staining solution (including 1 mg/ml DAB; 50 mM NaAc-HAc, pH = 3.8) for H_2_O_2_ or the NBT solution (including 1 mg/ml NBT; 25 mM Hepes, pH = 7.6) for superoxide, followed by vacuuming for 10 min. These samples were incubated for about 15 min to several hours (based on the staining degree) in the dark at 37 °C, and then the plants were transferred into 75% (v/v) ethanol to remove the chlorophyll.

### Reactive oxygen species measurement

The 7-day-old seedlings were transferred to clean filter paper and treated with 1/2 MS liquid culture with or without 100 mM NaCl for 24 h. The 0.1 g samples were frozen in liquid nitrogen, then the H_2_O_2_ and superoxide content was determined according to the H_2_O_2_ measuring kit (Solarbio) and superoxide measuring kit (Solarbio) protocols, respectively.

### RNA extraction and reverse transcription-quantitative polymerase chain reaction analysis

The total RNA of the seedlings was extracted using a Plant RNA Isolation Mini Kit (CWBIO). The RNA reverse transcription was performed using the HiScript II QRT Super mix for the qPCR (Vazyme Biotech), and the qPCR was performed using the SYBR Green Master Mix (Vazyme Biotech) and the iQ5 system (Bio-Rad). Three biological samples were analyzed with three separate technical replicates. *ACTIN* (AT3G18780) was used as a reference gene for normalization. The relative gene expressions were calculated using the 2^inu − ΔΔct^ method [55]. Three biological replicates were analyzed with three separate technical replicates. Error bars represent the SD from three biological replicates. The primers used for the RT-qPCR are listed in Supplemental Table 1.

### Western blotting

The seven-old-day seedlings were transferred to clean filter paper and treated with 1/2 MS liquid culture with or without 100 mM NaCl for 24 h. The total proteins were extracted, followed by 12% SDS/PAGE gel electrophoresis. The protein was transferred into a PVDF membrane (BioRad) by wet-tank transfer and detected using anti-GFP antibodies (Abmart). The antibody against ACTIN (Abmart) was used as the loading control. Quantitative analysis was performed by ImageJ software.

### Statistical analysis

The statistical data were analyzed with one-way ANOVAs (Tukey’s test, *P* < 0.05) and Mann- Whitney U test (*p* < 0.05) in SPSS16.0 (Polar Engineering and Consulting, http://www.winwrap.com). Different letters and star symbol were used to indicate statistically significant differences.

### Availability of data and materials

All the data and materials that are required to reproduce these findings can be shared by contacting the corresponding author, wangjuan@caas.cn (J.W.).

## Supplementary Information

**Additional file 1.** Supplemental Table 1

## Abbreviations

ABA: Abscisic acid
AsA: L-Ascorbic acid
APX: Ascorbate peroxidase
DAB: Diaminobenzidine
GFP: Geen fluorescent protein
MS: Murashige and skoog
NBT: Nitroblue tetrazolium
OE: Over-expression
qPCR: Quantitative polymerase chain reaction
ROS: Reactive oxygen species
