# Supplementary material for: Ascorbic acid modulation by ABI4 transcriptional repression of VTC2 in the salt tolerance of Arabidopsis
Source: BMC Plant Biol. 2021 Feb 24;21:112. doi: 10.1186/s12870-021-02882-1 (PMC7905542; doi:10.1186/s12870-021-02882-1)
Supplement: Supplementary file 1 — Additional file 1. Supplemental Table 1 [file 12870_2021_2882_MOESM1_ESM.docx]

**Supplemental Table 1. Primers used in this study**

| Primer name | Primer sequence (5' - 3' ) |
| --- | --- |
| AtABI4-F | CTTCCCAACATCAACACAAC |
| AtABI4-R | GGCATAACATAGAGGTCCCAC |
| AtVTC2-F | GCTTCCACCTTCAAAGAGAA |
| AtVTC2-R | CCAGTGTTGTTGCCATCA |
| qAtACTIN-F | GGCAAGTCATCACGATTGG |
| qAtACTIN-R | CAGCTTCCATTCCCACAAAC |
| qAtABI4-F | CGTTAGGGCAGGAACAAGGA |
| qAtABI4-R | ATAACCCGGATCCAGACCCA |
| qAtVTC2-F | TTCAGACTGCTGTGTTTGCCT |
| qAtVTC2-R | CAATACCTCCGGGCTCACTT |
| qAtVTC1-F | CAAAACCGGTCCAACACCAGA |
| qAtVTC1-R | CTGAGCTGGGAAATGGCTGTA |
| qAtVTC4-F | GGATGAGTGGTTCGTGTGCA |
| qAtVTC4-R | CTCAGCGAATAACTCCTTGA |
| qAtVTC5-F | AATGTGAGTCCGATTGAGTA |
| qAtVTC5-R | AGTAAGCCTGAAAGTGAAGA |
| qAtGalDH-F | ATTTCGTCGGTGTTGGTTGG |
| qAtGalDH-R | CCACTTGGCCATGTCAGATT |
| qAtGLDH-F | GCAGATTGGTGGTATTATTC |
| qAtGLDH-R | GACCTCAGCAACAACTCC |
| qAtAPX1-F | ATTGCTTAATGTACTCTCGGATTTACG |
| qAtAPX1-R | CAAAAGCGCAACGGATGTG |
| qAtAPX2-F | CTGGTGGACACACCTTGGG |
| qAtAPX2-R | GCAGCATATTTTTCAACAAATGGGA |
| qAtAPX3-F | GTTCCGTCGTCTTGTTGAGC |
| qAtAPX3-R | GTCTGCAACTGCTTTGCCTG |
| qAtAPX4-F | ACTGACCCTCAAGTTGCTCC |
| qAtAPX4-R | GCCTCGAAGTTGATTTGCTGT |
| qAtAPX5-F | GCATGATGCAGGAACCTATGATG |
| qAtAPX5-R | ACGGTTTAGCTCTTCCTTGAATCTG |
| qAtAPX6-F | CGGCCCAACAATTCCAGTAGT |
| qAtAPX6-R | GAGGTAGCTTGCCTTCTGGATC |
